# Annotations

**Published:** 1897-01-23

**Authors:** 


### ANNOTATIONS.

### The B.M.A. and Medical Defence.

THE committee of the British Medical Association specially appointed to consider the question of medical defence has just issued its first report. The most important point to be noticed in the report is that the committee declines entirely to commit the association as an association to the work of medical defence. What is proposed is that a third medical protection society, acting under the auspices of the British Medical Association, shall be formed. That such is the true significance of the report is proved by the following: Section 13 resolves, "That the accounts in the matter of medical defence be kept entirely distinct from the general accounts of the association." If this recommendation be considered in connection with section 4, it will be quite obvious that all the profession has to expect at the hands of the B.M.A. is a standing committee, entirely distinct from the association, having no financial or other organic connection with it; in fact, the addition of a third association for medical defence to the two already existing, Section 4 recommends, "That a standing committee consisting of nine members, five to form a quorum, be appointed to carry out the duties of medical defence, of whom not more than four may be members of the association who are not upon the Council." That such a standing committee, quite inexperienced in the work of medical defence and protection, will do its work better than either of the two independent and experienced bodies who are now carrying out medical protection and defence, is not to be expected. But that they may make the work of defence more costly, though not more efficient, is pretty plain. For it is provided that, not only shall there be a paid secretary, but also that now at once the members of the committee shall receive their travelling expenses; whilst in future it is a recommendation that they shall also be paid for their services. The profession will do well to give these proposals its serious consideration; and to compare them with the articles of association and the bye-laws of the existing protection and defence societies.

### A National Medical Club.

THE most effectual method of putting an extinguisher upon medical aid societies and other similar institutions would certainly be to provide something better in their places. "Beta," writing to a medical contemporary, proposes to substitute a National Medical Club, instituted and controlled largely by the medical profession. The Oddfellows and Foresters, he argues, provide certain benefits for their members, and manage to thrive. Why should not a national association of medical men, either directly or by the agency of an assurance office, provide similar benefits all over the country, and thrive likewise? Working men need, we are told, first of all, medical attendance at a cheap rate; and this can be provided for them at a penny a week, or four shillings per annum. In addition they need sick pay to the amount of ten or twelve shillings per week; and this also can be provided for by a weekly subscription of about fivepence. That is to say, for sixpence a week, sick pay and medical attendance of the best quality can be furnished to *bonâ-fide* working men. Two more conditions require to be fulfilled, the first being that the National Medical Club shall be founded on so sound a general and financial basis as to ensure its permanency, and make every member feel that it will certainly "last his lifetime"; and the second that care should be taken to establish branches practically "everywhere" throughout the country, so that a working man moving from place to place might, so to speak, carry his club and all its benefits with him. This seems to us to be really an excellent proposal; and if it could be carried out on sound principles as to business management, the appointment of medical officers, and the like, there would be good ground for hoping that in due time the National Medical Club would supersede most of the institutions of its kind. Of course, there are many pros and cons to be considered, and four bald suggestions do not constitute a working scheme; but the general idea appears to be well worthy of the consideration of the shrewdest heads in general practice.

### The Causes of Disease.

PROFESSOR JACCOUD, in a clinical lecture delivered at the Pitié Hospital, urges that in the consideration of microbial diseases the non-microbial factors in their causation should never be lost sight of. He points out what is perfectly well recognised by all authorities, that although many diseased conditions are the result of the growth of microbes within the body of the patient, such growth often depends on the condition of the individual allowing them to develop, rather than on any overmastering power of intrusion possessed by the micro-organism themselves. He urges also that in many cases the time of the development of the disease bears no relation to the moment when the microbe gained access, the sinning organism being in some cases a permanent denizen of the body, and only becoming pathogenic in consequence of some change in the constitution of the patient. From this he argues that the virus is only one of the factors in the development of disease, and that it does not take effect unless the patient is in a receptive state. This predisposition may be innate and permanent, or it may be accidental and temporary, and it constitutes the morbid opportunity without which the virus remains inactive, or at most determines slight disorders quite different from those it produces when in full activity. We thus have brought back to us the importance attributed by all the older race of physicians to congenital predisposition, constitution, temperament, and even to so-called "cosmic influences," all of which must be looked upon as factors in the production of disease, just in so far as they render the organism more fitting soil for the growth of the micro-organism within it. These ideas are old enough, and are generally recognised by those who have studied the etiology of disease. Nevertheless, it is well that they should be repeated and enforced time after time; for the idea that infectious diseases depend solely upon infection is extremely seductive, and has peculiar fascinations for half-educated sanitary authorities. Natural science refuses, however, to be limited to such simple hypotheses, and those who would understand the causes of infectious diseases must understand that under the term "causes" are included not merely the microbe, but all the details in the social life of to-day which render man more or less open to its attacks. This is a long story, and far from simple.

